# Supplementary material for: Causal Relationship between Aging and Anorexia Nervosa: A White-Matter-Microstructure-Mediated Mendelian Randomization Analysis
Source: Biomedicines. 2024 Aug 16;12(8):1874. doi: 10.3390/biomedicines12081874 (PMC11351342; doi:10.3390/biomedicines12081874)
Supplement: Supplementary file 1 [file biomedicines-12-01874-s001.zip › Supplementary Figure/Figure S4/Singe SNP plot of MR analysis for TL to ubm-b-1859.pdf]

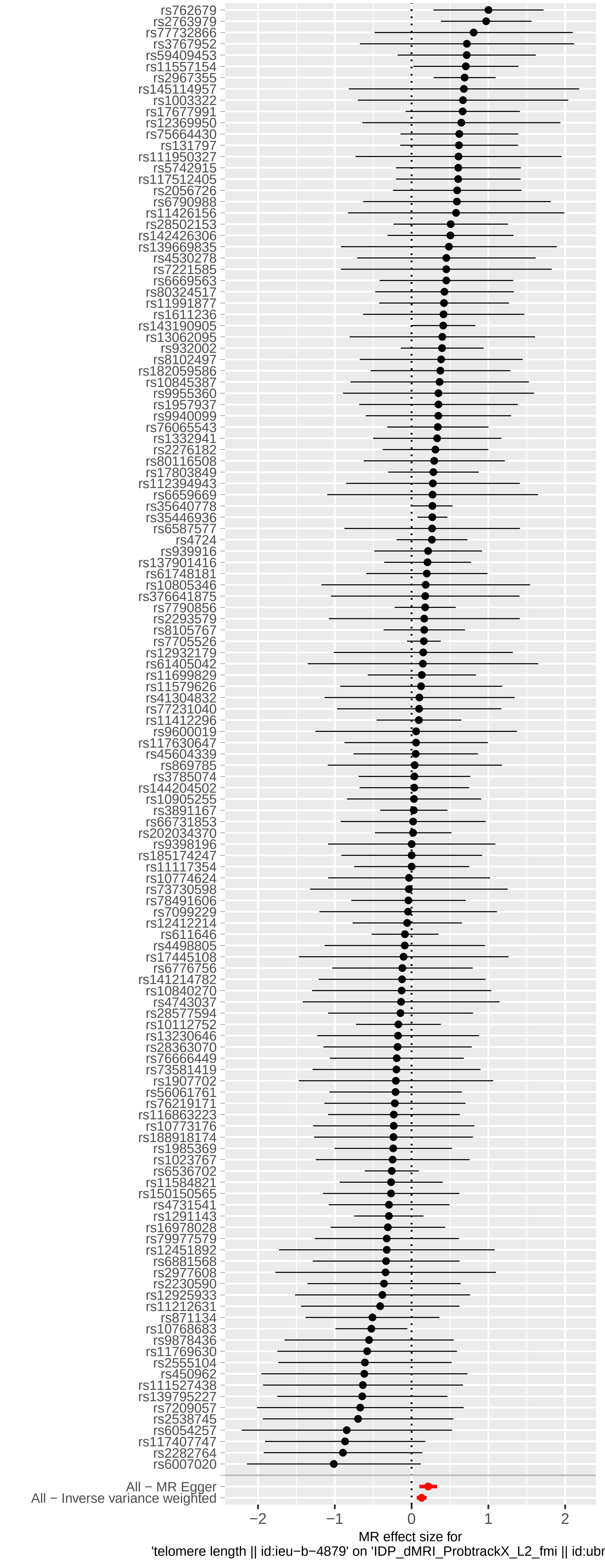
rs762679
rs2763979
rs77732866
rs3767952
rs59409453
rs11557154
rs2967355
rs145114957
rs1003322
rs17677991
rs12369950
rs75664430
rs131797
rs111950327
rs5742915
rs117512405
rs2056726
rs6790988
rs11426156
rs28502153
rs142426306
rs139669835
rs4530278
rs7221585
rs6669563
rs80324517
rs11991877
rs1611236
rs143190905
rs13062095
rs932002
rs8102497
rs182059586
rs10845387
rs9955360
rs1957937
rs9940099
rs76065543
rs1332941
rs2276182
rs80116508
rs17803849
rs112394943
rs6659669
rs35640778
rs35446936
rs6587577
rs4724
rs939916
rs137901416
rs61748181
rs10805346
rs376641875
rs7790856
rs2293579
rs8105767
rs7705526
rs12932179
rs61405042
rs11699829
rs11579626
rs41304832
rs77231040
rs11412296
rs9600019
rs117630647
rs45604339
rs869785
rs3785074
rs144204502
rs10905255
rs3891167
rs66731853
rs202034370
rs9398196
rs185174247
rs11117354
rs10774624
rs73730598
rs78491606
rs7099229
rs12412214
rs611646
rs4498805
rs17445108
rs6776756
rs141214782
rs10840270
rs4743037
rs28577594
rs10112752
rs13230646
rs28363070
rs76666449
rs73581419
rs1907702
rs56061761
rs76219171
rs116863223
rs10773176
rs188918174
rs1985369
rs1023767
rs6536702
rs11584821
rs150150565
rs4731541
rs1291143
rs16978028
rs79977579
rs12451892
rs6881568
rs2977608
rs2230590
rs12925933
rs11212631
rs871134
rs10768683
rs9878436
rs11769630
rs2555104
rs450962
rs111527438
rs139795227
rs7209057
rs2538745
rs6054257
rs117407747
rs2282764
rs6007020
All − MR Egger
All − Inverse variance weighted
−2
−1
0
1
2
MR effect size for
'telomere length || id:ieu−b−4879' on 'IDP_dMRI_ProbtrackX_L2_fmi || id:ubr
